# Electrophysiological Evidence of Enhanced Processing of Novel Pornographic Images in Individuals With Tendencies Toward Problematic Internet Pornography Use

**DOI:** 10.3389/fnhum.2022.897536

**Published:** 2022-06-23

**Authors:** Jianfeng Wang, Yuanyuan Chen, Hui Zhang

**Affiliations:** School of Psychology, Chengdu Medical College, Chengdu, China

**Keywords:** problematic internet pornography use, novelty seeking, late positive potential, event-related potentials, addiction

## Abstract

Novelty seeking is regarded as a core feature in substance use disorders. However, few studies thus far have investigated this feature in problematic Internet pornography use (PIPU). The main aim of the present study was to examine group differences in electrophysiological activity associated with novelty processing in participants with high tendencies toward PIPU vs. low tendencies using event-related potentials (ERPs). Twenty-seven participants with high tendencies toward PIPU and 25 with low tendencies toward PIPU completed a modified three-stimulus oddball task while electroencephalogram (EEG) was recorded. Participants were instructed to detect neutral target stimuli from distracting stimuli. The distracting stimuli contained a familiar sexual stimulus and a set of novel sexual stimuli. The novel-familiar difference waves were calculated to identify specific group difference in novelty effect. While both groups demonstrated a sustained novelty effect in the late positive potential (LPP) within the 500–800 ms time windows, the novelty effect was greater in the high tendencies toward PIPU group than in the low tendencies toward PIPU group. This result suggests that individuals with high tendencies toward PIPU allocate more attentional resources for novelty processing. Enhanced brain responding to novel sexual stimuli may facilitate pornographic consumption and play an essential role in the development and maintenance of PIPU.

## Introduction

Problematic Internet pornography use (PIPU) refers to the phenomenon that people are unable to control their overuse of Internet pornography despite its negative consequences (Wéry and Billieux, 2017). The spread of the Internet has led to a dramatic increase in online porn consumption over the past few decades (Kohut et al., 2020). About 87% of men watch pornography monthly, and about 58% watch it weekly (Brem et al., 2018). Epidemiological surveys estimate the prevalence of sexual addiction to be between 0.1 and 5% for women and 2–13% for men (Ballester-Arnal et al., 2017; Rissel et al., 2017).

So far, there is no consensus on the classification and diagnosis of PIPU. In 2018, compulsive sexual behavior disorder (CSBD) was classified in the impulse control disorder chapter of the ICD-11. While people may suffer from various forms of compulsive sexual behavior, the most prominent behavior is viewing pornography. Therefore, PIPU has been considered a manifestation of CSBD (Kraus et al., 2018). According to the definition of impulse control disorder, which is characterized by an inability to control behavior despite its negative effects, the ICD-11 Task Force proposed that CSBD be included in this group. However, there are also many scholars suggesting that its classification may be more appropriate as a behavioral addiction rather than an impulse control disorder (e.g., Stark et al., 2018). Accumulating evidence suggests that PIPU is analogous to drug addiction or other behavioral addictions (Seok and Sohn, 2015; Kowalewska et al., 2018; Stark et al., 2018). For example, PIPU is related to cue-reactivity for sex-related stimuli (Brand et al., 2011; Voon et al., 2014; Wang et al., 2021), and this mechanism is also present in the development and maintenance of substance-related addictions (e.g., Skinner and Aubin, 2010). Moreover, neural activation toward sex- and drug-related stimuli involves similar brain networks, presumably modulated by the mesolimbic dopamine circuits (Brand et al., 2016; Gola and Draps, 2018).

Novelty seeking, as both a cause and a symptom of addiction, is known to exert an essential effect on the development and maintenance of addiction. Novelty seeking is defined as the tendency to desire novel stimuli and environments (Zuckerman, 1994; Wingo et al., 2016). Substantial evidence has revealed that high novelty seeking increases susceptibility to behavioral and substance addictions (Foulds et al., 2016; Wingo et al., 2016). For example, in animal studies, novelty responses are related to compulsive cocaine use (Belin and Deroche-Gamonet, 2012). Similar findings have also been observed in humans, suggesting that novelty seeking is intimately linked to propensity to and relapse of addiction (Ismael and Baltieri, 2014; Mahoney et al., 2015). An important reason for the strong relationship between the two lies in the fact that the same neurotransmitter and neural circuitry are activated by the novelty and drugs of abuse (Bardo et al., 1996; Wingo et al., 2016). Specifically, both novelty seeking and addictions are regulated by the mesocorticolimbic dopaminergic systems. As occurs with exposure to drugs of abuse, exposure to novel stimuli increases dopamine release in the nucleus accumbens (Bardo et al., 1996; Morrens et al., 2020).

Pornography has addictive qualities, but it doesn’t take the exact same form as other substance addictions (Zimbardo and Coulombe, 2015). With tobacco, alcohol, or drugs, people want more of the same thing. But with pornography, people need a lot of novelty and wacky stuff to get the same thrill. In animal studies, researchers have found a phenomenon known as the Coolidge effect, in which introducing a new mate to sexually satisfied males restore their sexual interest and mating activity (Dewsbury, 1981). Similarly, some studies have found that men prefer sexual novelty and show greater sexual arousal to novel sexual stimuli. For example, Koukounas and Over (2000) found that repeated exposure to pornographic film clip led to a progressive decline in sexual arousal, while substituting the repetitive pornographic movie with a novel pornographic movie restored sexual arousal. Joseph et al. (2015) have found that masturbating to a novel pornstar increases ejaculate volume and motile sperm compared with masturbating to a familiar actress. Also, time to ejaculation decreased in response to the novel women. In addition, sex differences in sexual novelty preferences were also revealed. Compared with females, males showed a greater preference for sexual variety during potential short-term relationships (Hughes et al., 2021).

Today’s Internet is increasingly filled with R-rated photos and videos. Many porn sites have compiled tens of thousands of tags to make it easier for users to find unusual content that suits their particular tastes. Internet porn is addictive precisely because it offers an endless supply of sexual novelty. To date, there has been very little research examining sexual novelty preference in individuals with PIPU. To our knowledge, only one study examined novelty preference in people with compulsive sexual behaviors (CSB) using behavioral measure (Banca et al., 2016). After participants became familiar with the pornographic and neutral pictures, they were asked to choose between a familiar or novel choice randomly associated with winning. It turns out that CSB participants preferred the novel over the familiar pornographic pictures’ options (Banca et al., 2016).

In this research, we investigated the potential effects of PIPU on brain processing of novel sexual images utilizing event-related potentials (ERPs). Prior research has shown that ERP is an effective tool for the objective assessment of sexual arousal and sexual interest in both healthy and clinical populations (Vardi et al., 2006; Ortigue et al., 2009; Steele et al., 2013; Ziogas et al., 2020). ERPs can assess the time course of neuronal activity and are a suitable tool to investigate the cognitive processing of sexual stimuli in the brain. ERPs refer to time-locked electroencephalography (EEG) when responding to stimuli. Different ERPs components reveal different phases of information processing and attentional involvement. ERPs studies of emotional processing have primarily concentrated on the late positive potential (LPP), an extended positivity with central-parietal sources that is very sensitive to motivationally relevant cues (Schupp et al., 2000; Hajcak and Foti, 2020). Compared to the P300, this later and more sustained component seems more sensitive to motivational processes (Schupp et al., 2000; Prause et al., 2015a). The LPP is increased when responding to emotionally evocative stimuli compared with neutral stimuli (e.g., Ito et al., 1998). Erotic images induced larger positive amplitudes than high arousal sport images, especially in late (500–750 ms) stage (van Lankveld and Smulders, 2008). A recent meta-analysis found a significant large effect size for the association between sexual stimulation and the LPP (Ziogas et al., 2020). Moreover, the enhancement of the LPP to addiction-related cues has also been well documented (e.g., Littel and Franken, 2007; Thalemann et al., 2007).

Classically the LPP or P300 is induced by using the oddball task in which frequent standard stimuli and infrequent deviant stimuli are presented. The present study used a modified three-stimulus oddball task, in which participants were instructed to identify target stimuli from distracting stimuli (see Yuan et al., 2012). The distracting stimuli contained a familiar sexual stimulus and a collection of novel sexual stimuli. The target stimuli (50% of trials, a portrait image) and the familiar stimulus (25%, a pornographic image) remained constant, while the novel stimulus (25%) was drawn without replacement from a set of pornographic images. With this approach, ERP differences between familiar and novel conditions should reflect novelty processing (Yuan et al., 2012).

Based on prior studies (e.g., Banca et al., 2016), we hypothesized that individuals with high tendencies toward PIPU would exhibit enhanced processing of novel vs. familiar sexual images compared with the low tendencies toward PIPU group. Specifically, we computed the novel-familiar difference waves by subtracting ERP to familiar stimuli from those for novel stimuli to acquire ERP directly reflecting novelty effect. We predicted that participants with high tendencies toward PIPU would display larger amplitude than the low tendencies toward PIPU participants in the difference wave, indicating increased brain responding to novel sexual images.

## Materials and Methods

### Participants

Our experiment sample size was set according to a prior power analysis using G*Power 3.1.9 (Faul et al., 2009). Based on medium effect size (*f* = 0.25), power (1-β error probability) of 0.80, and α error probability of 0.05, the power analysis yielded an estimated sample size of 34. A total of 263 male college students were recruited to complete the Problematic Internet Pornography Use Scale (PIPUS; Kor et al., 2014; Chen and Jiang, 2020). Women were excluded from the study due to PIPU is more prevalent among men (e.g., Pekal et al., 2018). Currently, PIPU is not officially recognized as a mental disorder, and there are no universally accepted diagnostic criteria. Thus, participants were selected based on the top and bottom quartiles of PIPUS scores (e.g., Wang et al., 2021). According to this classification, 60 participants (30 in high tendencies toward PIPU group and 30 in low tendencies toward PIPU group) volunteered to attend the experiments. Exclusion criteria were age younger than 18 years, having a history of substances abuse, and having psychiatric diseases including depression and obsessive-compulsive disorder. Eight participants (three from the high tendencies toward PIPU group and five from the low tendencies toward PIPU group) were excluded because of equipment problems and excessive eye movement artifacts.

### Materials and Procedure

PIPU was measured by the Chinese version of the PIPUS (Chen and Jiang, 2020). The scale consists of 12 items measuring 4 dimensions of PIPU, namely suffering and functional impairments, excessive use, loss of control, and avoidance of negative emotion. Items are rated on a 6-point scale from 0 (*never*) to 5 (*always*). The Cronbach’s α for this sample was 0.98.

First, participants were asked to fulfill the PIPUS. According to the above classification method, 60 participants voluntarily participated in the three-stimulus oddball task and their EEG activity was picked up simultaneously. After the task was completed, participants needed to fulfill a set of questionnaires including Barratt Impulsiveness Scale-11 (BIS-11; Patton et al., 1995; α = 0.90), Obsessive-Compulsive Inventory-R (Foa et al., 2002; α = 0.90), Self-Rated Depression Scale (Zung et al., 1965; α = 0.83), and Self-Rated Anxiety Scale (Zung, 1971; α = 0.84) to assess impulsivity, obsessive-compulsive symptoms, depression, and anxiety, respectively. In addition, each participant completed an additional task unrelated to this study. Participants were thanked and paid for taking part.

### Stimuli and Experimental Task

A modified three-stimulus oddball task was adopted in this experiment. The experiment included 2 blocks. Each block consisted of 40 target (50% probability), 20 familiar (25% probability), and 20 novel stimuli (25% probability). The probability of target stimuli and non-target stimuli are equal, thus avoiding the influence of inhibitory control when non-target stimuli occur infrequently (Yuan et al., 2008). The target stimulus was a portrait image, the familiar stimulus was a pornographic image, and the novelty stimuli were 20 non-repeated pornographic images. The novel stimuli in the two blocks were not repeated, while the familiar stimulus was the same. All pornographic images, depicting penile-vaginal intercourse between a male and a female, were obtained from free available pornography sites. All pornographic images were assessed in a pilot study, showing no significant differences between familiar and novelty stimuli in valence, arousal, and sexual arousal dimensions.

Participants were tested in a sound-attenuated room approximately 100 cm from a computer monitor. Both the horizontal and vertical visual angles were less than 6°. The experimental task includes the familiarization stage and the testing stage. In the familiarization stage, four pornographic pictures were presented in pairs to the participants, resulting in 12 trials. Each trial was presented for 10 s, during which the participants were asked to carefully observe the images. We randomly selected one of the four images as the familiar stimulus.

In the testing phase (three-stimulus oddball task), each trial was initiated with a fixation cross for 300 ms, followed by the presentation of a blank screen for 500–1,000 ms. Afterward, pictures were presented in randomized order. Participants were instructed to detect the target stimuli by pressing the spacebar, in a series of distracting stimuli (both familiar and novel stimuli) that should be ignored. The picture vanished after the response was made or 1,000 ms, followed by a blank screen for 1,000 ms. All images were in color and presented in the center of a computer screen against a black background.

### Electrophysiological Recording and Analysis

EEG was recorded by Ag/AgCl electrode cap using the 64-channel EEG system of Brain Product (Brain Products GmbH, Munchen, Germany). An electrode was placed below the right eye to record the vertical electroencephalograms (EOGs), and a ground electrode was located at the midpoint of the connection between FPz and Fz electrodes in the anterior middle of the scalp. The sampling rate was 500 Hz, and the electrode impedances were less than 5 kΩ. Continuous EEG data were analyzed offline with Brain Vision Analyzer version 2.0. FCz electrode was used as online reference whereas the left and right mastoid electrodes were averaged to serve as offline reference. Data were band-pass filtered between 0.01 and 30 Hz (slope: 24 dB/oct). ERP epochs were extracted beginning 200 ms before and ending 800 ms after sexual stimuli presentation, and trials were accepted only if the answer of both the familiar and the novel stimuli were correct (i.e., no keystroke response was made). The 200 ms pre-stimulus was used as baseline to each epoch. Thereafter, ocular artifacts were corrected using independent component analysis. Epochs with voltages exceeding ± 80 μV (relative to baseline) at any electrode were excluded from analysis. Mean trials for familiar and novel conditions were 37.75 (*SD* = 4.17) and 37.82 (*SD* = 4.02), respectively.

**FIGURE 1:**
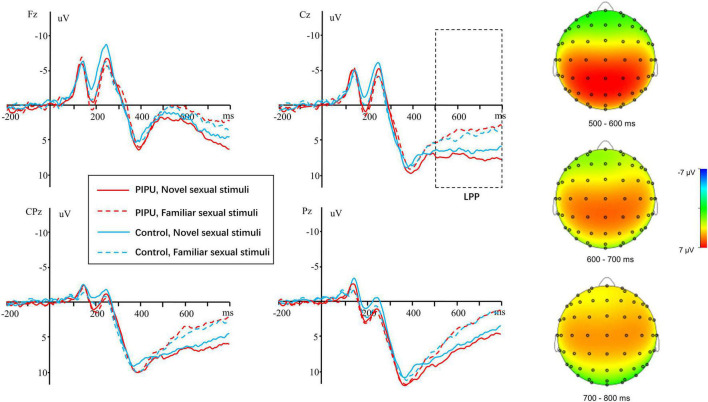
The grand average and scalp topography of LPP component for the individuals with high tendencies toward PIPU vs. low tendencies under the novel and familiar sexual stimuli for electrodes sites at Fz, Cz, CPz, and Pz.

EEG signals in the familiar and the novel conditions were aligned and averaged separately. The LPP was defined as the average amplitude at the electrodes of F3, Fz, F4, FC3, FCz, FC4, C3, Cz, C4, CP3, CPz, CP4, P3, Pz, and P4 between 500 and 800 ms. The Electrodes and time window were chosen according to previous literature (van Lankveld and Smulders, 2008; Prause et al., 2015b; Wang and Dai, 2020) and confirmed with visual inspection of the grand average waveforms (see Figure 1).

### Statistical Analysis

The questionnaire and behavior data were analyzed by independent sample *t*-test. For the LPP amplitudes, a 2 × 2 × 15 repeated measures analysis of variance (ANOVA) was conducted with electrode and condition (familiar vs. novel) as within-subject factors and group (high tendencies toward PIPU vs. low tendencies toward PIPU) as a between-subject factor. If significant interaction between group and condition was observed, we performed simple effects tests comparing novel vs. familiar images, separately for high and low tendencies toward PIPU. For these planned comparisons, the criterion of the *p*-value was set at 0.05/2 = 0.025 (Bonferroni-corrected). Greenhouse–Geisser corrections were reported. For ANOVA and *t*-test, effect sizes were expressed as partial eta square (η^2^_*p*_) or Cohen’s *d*, respectively.

### Ethics

The study was in accordance with the Declaration of Helsinki. All participants understood the contents of the experiment and provided informed consent. This study was approved by the local ethical committee.

## Results

### Questionnaire Results

Questionnaire variables were presented as the mean and standard deviation (*SD*) in Table 1. PIPUS scores of the PIPU group was significantly higher than that of the control group. In addition, the PIPU group had significantly higher SAS and BIS-11 scores than the control group.

**TABLE 1:** Descriptive statistics of the participants with high tendencies toward PIPU vs. low tendencies on questionnaires.

Variable (M ± *SD*)	High tendencies toward PIPU (*n* = 27)	Low tendencies toward PIPU (*n* = 25)	*t*	*p*	*d*
Age (years)	18.63 ± 1.01	19.04 ± 1.70	1.07	0.289	0.30
PIPUS	36.96 ± 14.96	12.40 ± 0.65	8.20	<0.001	2.28
SDS	36.48 ± 10.71	32.76 ± 10.09	1.29	0.204	0.36
SAS	37.11 ± 9.87	30.24 ± 7.40	2.82	0.007	0.78
BIS-11	76.78 ± 10.33	69.04 ± 12.54	2.44	0.018	0.68
OCI-R	37.41 ± 10.40	36.12 ± 12.73	0.40	0.690	0.11

*PIPUS, Problematic Internet Pornography Use Scale; SDS, Self-Rated Depression Scale; SAS, Self-Rated Anxiety Scale; BIS-11, Barratt Impulsiveness Scale-11; OCI-R, Obsessive-Compulsive Inventory-R.*

### Behavioral Results

Independent sample *t*-test revealed no significant main effects of group on reaction times (RTs) and accuracy (see Table 2). All participants were able to detect target stimuli quickly (*M* = 231.50 ms) and accurately (*M* = 99.65%).

**TABLE 2:** Descriptive statistics for accuracy and RTs measures for the target identification for each group.

Variable (M ± *SD*)	High tendencies toward PIPU (*n* = 27)	Low tendencies toward PIPU (*n* = 25)	*t*	*p*	*d*
ACC (%)	99.67 ± 0.68	99.64 ± 0.86	0.13	0.901	0.04
RTs (ms)	237.88 ± 37.69	224.62 ± 31.15	1.38	0.175	0.38

*ACC, accuracy rate; RTs, reaction times; PIPU, Problematic Internet Pornography Use.*

### Event-Related Potentials Results

**FIGURE 2:**
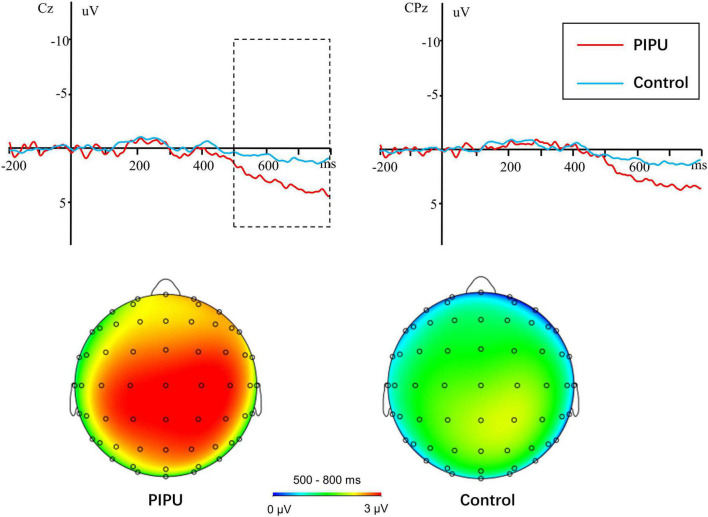
Novel-familiar difference waves in individuals with high tendencies toward PIPU vs. low tendencies and their topographical maps during the 500–800 ms time windows.

For the LPP amplitudes, analyses were conducted by using repeated measures ANOVA. The outcomes of these ANOVAs are shown in Table 3. There was a significant main effect of condition [*F*(1, 50) = 41.14, *p* < 0.001, η^2^_*p*_ = 0.45], with novel stimuli (5.28 ± 3.38 μV) evoking larger positive amplitudes than the familiar stimulus (3.20 ± 3.06 μV). A significant main effect of electrode [*F*(14, 700) = 18.86, *p* < 0.001, η^2^_*p*_ = 0.27] and a condition × electrode interaction [*F*(14, 700) = 2.76, *p* = 0.049, η^2^_*p*_ = 0.05] was also observed. The amplitude differences between novel and familiar stimuli were more prominent at central and parietal sites than at frontal sites (all *p*s < 0.05). Importantly, there was a significant group × condition interaction [*F*(1, 50) = 6.29, *p* = 0.015, η^2^_*p*_ = 0.11).^1^ The simple-effect of condition (Bonferroni corrected *p* = 0.025) revealed that novel stimuli (5.58 ± 3.30 μV) evoked larger amplitudes compared with familiar stimuli (2.68 ± 2.90 μV) in the high tendencies toward PIPU [*F*(1, 26) = 57.74, *p* < 0.001, η^2^_*p*_ = 0.69], but there was only a marginally significant difference in amplitude between stimuli (4.98 ± 3.51 vs. 3.71 ± 3.19 μV) for the low tendencies toward PIPU [*F*(1, 24) = 5.62, *p* = 0.026, η^2^_*p*_ = 0.19] groups (see Figure 2).

**TABLE 3:** Outcome of ANOVAs of mean amplitudes of LPP.

	*df*	*F*	*p*	η^2^_*p*_
Condition	1, 50	41.14	<0.001	0.45
Electrode	14, 700	18.86	<0.001	0.27
Condition × electrode	14, 700	2.76	=0.049	0.05
Group × condition	1, 50	6.29	=0.015	0.11

### Additional Analyses

We also examined the early P2 (150–220 ms) and N2 (220–300 ms) components. There were no other significant interaction effects except for increased negative deflection recorded during novel than during familiar conditions (all *p*s < 0.01). Given that no interaction between group and condition was present and the early components are less consistently related to emotion and attentional processing (de Rover et al., 2012; Sabatinelli et al., 2013), these findings are considered preliminary and not pursued further here.

## Discussion

The present study examined the differences in the novelty processing of pornographic pictures between participants with high tendencies toward PIPU vs. low tendencies, using a three-stimulus oddball task based on electrophysiological indices. We found a significant ERPs effect in the 500–800 ms interval (i.e., the LPP effect). This finding demonstrated that enhanced brain processing of sexually stimuli novelty in individuals with high tendencies toward PIPU compared to the low tendencies toward PIPU. To our knowledge, this is the first electrophysiological evidence of enhanced novelty processing to sexual stimuli in the domain of pornography addiction.

In the time window of 500–800 ms, we observed a prominent sustained positivity activity, and its amplitudes were most pronounced at the midline central-parietal recording sites, which fits with the morphology of the attention-related LPP from prior emotion studies (Hajcak and Foti, 2020). Generally, LPP is thought to be involved in motivational attention and meaning evaluation of stimuli (Johnson, 1993; Hajcak and Foti, 2020). The amplitude is increased with stimulus significance (Hajcak and Foti, 2020). Moreover, LPP is vulnerable to probability (Johnson, 1993) and pre-motor response preparation (Schupp et al., 2000). Because both novel and familiar stimuli had the same probability and were task-unrelated distracting stimuli that required no key-presses, the LPP difference between novel and familiar sexual stimuli reflected solely novelty-related cognitive processing. In the present work, a larger LPP amplitudes in response to novel sexual stimuli than to familiar sexual stimuli was observed for both samples. This observation is in agreement with the previous studies which showed that men were more likely to desire for sexual novelty (D’Orlando, 2011). For example, when the participants were shown the same erotic film repeatedly, their sexual arousal progressive decreased. But when a new erotic film was introduced, their sexual arousal dramatically increased (Koukounas and Over, 2000). Similarly, masturbating to a novel pornstar increases ejaculate volume and motile sperm compared with masturbating to a familiar actress (Joseph et al., 2015). It has been suggested that the decrease in sexual arousal is due to pornographic stimuli becoming less attention-demanding with repetition. In a study conducted by Koukounas and Over (1999), they assessed RTs to a secondary-task probe (tone) displayed intermittently while men were watching the pornographic movie clip. Decreased sexual arousal due to repetition was accompanied by a faster response to tone. Conversely, increased sexual arousal due to the introduction of a novel erotic film clip resulted in a slower response to tone. Such results suggest that habituation of sexual arousal is accompanied by a transfer of attentional resources. Our results further corroborate the findings of Koukounas and Over (1999) with electrophysiological evidence.

In addition to the significant novelty effect in both groups, we also found that the novelty effect was greater in the high tendencies toward PIPU group than in the low tendencies toward PIPU group. This suggests that individuals with high tendencies toward PIPU allocate more attentional resources for novelty processing. This finding is consistent with a previous behavioral study reporting that CSB individuals exhibited enhanced novelty preference for sexual images relative to healthy volunteers (Banca et al., 2016). Additionally, the enhanced novelty processing to sexual stimuli in individuals with high tendencies toward PIPU is also congruent with those of previous studies that indicated novelty seeking was related to substance use disorder (Bardo et al., 1996; Arenas et al., 2016; Wingo et al., 2016). Extensive evidence suggests that novelty seeking predicts the propensity and severity of disorders of addiction. For example, novelty seeking during adolescence is thought to predict a risk of alcohol, nicotine, cannabis, and other substance use disorders in adulthood (Palmer et al., 2013; Foulds et al., 2017). In Parkinson’s patients with impulse control disorders, novelty seeking is linked to pathological gambling and compulsive shopping (Voon et al., 2011). Similarly, both people with behavioral addictions and those with substance use disorders scored high on self-reported measures of sensation-seeking (Kim and Grant, 2001; Kelly et al., 2006). Therefore, these findings indicate that, with respect to novelty seeking, PIPU is very similar to substance use disorder or other behavioral addictions. Our results support the claim that PIPU can be considered as a behavioral addiction (e.g., Brand et al., 2014; Wang and Dai, 2020). In DSM-5 and ICD-11, gambling disorder has moved from impulse control disorders to addiction disorders. Further research is required to establish the most appropriate classification of PIPU (and more generally CSBD).

Our results may be particularly meaningful in the context of the high prevalence of pornography consumption. The Internet is increasingly saturated with pornographic images and videos, providing an endless source of novelty. For example, even though 5% of the tags cover 90% of the content, one popular porn site edited more than 70,000 tags to help users find novel or unusual porn content (Mazières et al., 2014). In the porn world, it’s easy to get used to repetition. Only something different keeps people focused. When excessive porn viewing becomes addictive, people need novel, more hard-core and stranger stimuli to be aroused.

Limitations to this study should be noted. First, this study did not include women, as PIPU is more prevalent among men. However, earlier studies have shown that women also show a novel preference for erotic stimuli (e.g., Meuwissen and Over, 1990). Therefore, it is necessary to investigate whether similar electrophysiological markers of novelty seeking are also present in women. Second, only young college students were recruited in this study, which may limit the generalizability of the findings. Future studies need to confirm the presented results in clinical samples of patients addicted to Internet pornography. Third, in this study, we found that individuals with high tendencies toward PIPU showed enhanced processing of novel sexual stimuli, but it is not clear whether enhanced novelty processing is a cause or a consequence of PIPU. Novelty seeking may be both a cause and consequence of PIPU. Further research is needed to confirm the actual causal mechanism.

## Conclusion

In conclusion, the current research provides initial electrophysiology evidence that individuals with high tendencies toward PIPU may exhibit enhanced brain responding to novel sexual stimuli. This heightened novelty effect may contribute to pornographic consumption and play an essential role in the development and maintenance of PIPU.

## Data Availability Statement

The raw data supporting the conclusions of this article will be made available by the authors, without undue reservation.

## Ethics Statement

The studies involving human participants were reviewed and approved by Chengdu Medical College Institutional Review Board. The patients/participants provided their written informed consent to participate in this study.

## Author Contributions

JW, YC, and HZ were involved in study conception and design. JW and YC were involved in data preparation, statistical analysis, and wrote the manuscript. JW and HZ were involved in study supervision and edited the manuscript. All authors had full access to all data in the study and take responsibility for the integrity of the data and the accuracy of the data analysis.

## Conflict of Interest

The authors declare that the research was conducted in the absence of any commercial or financial relationships that could be construed as a potential conflict of interest.

## Publisher’s Note

All claims expressed in this article are solely those of the authors and do not necessarily represent those of their affiliated organizations, or those of the publisher, the editors and the reviewers. Any product that may be evaluated in this article, or claim that may be made by its manufacturer, is not guaranteed or endorsed by the publisher.

## Footnotes

1 Because significant group differences in BIS-11 and SAS scores were observed, we conducted further analyses including BIS-11 and SAS scores as covariates to remove the effects of impulsivity and anxiety. The interaction between group and condition remained significant when these two variables were included as covariates in statistical analyses, *F*(1, 48) = 4.27, *p* = 0.044, η^2^_*p*_ = 0.08.
